# What Is the Contribution of Maternal BMI to the Risk of Adverse Pregnancy Outcomes?

**DOI:** 10.1111/ajo.70163

**Published:** 2026-07-27

**Authors:** Megan Mitchell, Andrea R. Deussen, Amanda J. Poprzeczny, Laura J. Slade, Jennie Louise, Jodie M. Dodd

**Affiliations:** ^1^ Adelaide University, School of Medicine the Robinson Research Institute Adelaide South Australia Australia; ^2^ Department of Obstetrics and Gynaecology The Women's and Children's Hospital, Women's and Babies Division Adelaide South Australia Australia; ^3^ Women's and Children's Hospital Research Centre Adelaide South Australia Australia; ^4^ Biostatistics Unit South Australian Health and Medical Research Institute Adelaide South Australia Australia

**Keywords:** body mass index, overweight and obesity, parity, pregnancy outcomes, risk

## Abstract

**Background:**

The associations between increased maternal body mass index (BMI) in pregnancy and adverse pregnancy outcomes for women and their infants are well known. Describing and understanding risk accurately can be problematic for women and clinicians, with a true picture often obscured by common reporting practices such as categorisation of BMI and use of only relative measures of risk.

**Aims:**

The aims were to investigate relationships between maternal BMI, parity, and adverse pregnancy outcomes; provide estimates of both relative and absolute risks of adverse pregnancy outcomes; and consider the potential contribution from unmeasured confounding.

**Materials and Methods:**

Data from the South Australian Perinatal Outcomes unit from 2013 to 2017 inclusive were analysed for associations between BMI categories, BMI as a continuous variable, parity, and adverse pregnancy outcomes.

**Results:**

Data from 65,285 women were included. A 5 kg/m^2^ increase in maternal BMI was associated with an increased adjusted relative risk (aRR) of gestational diabetes between 1.3 and 1.4 times, though the adjusted risk difference (aRD) was just 3%, irrespective of parity. The aRR of pre‐eclampsia was 1.13 times and 1.43 times in nulliparous and multiparous women respectively; however, the aRD was less than 0.1% for all women. The absolute risk of pre‐eclampsia at a BMI of 20 was 0.7% for nulliparous and 0.2% for parous women, which increased to a risk of 0.9% for nulliparous and 0.4% for parous women.

**Conclusions:**

While risks of most adverse outcomes increase with increased BMI, we cannot rule out the possibility that a substantial amount of the observed effects are due to unmeasured confounding. Use of BMI categories leads to underestimation of the true risk for some women and overestimation for others. Use of only relative measures of risk may create an impression that the risks associated with BMI are more dramatic than the absolute risks would suggest. Additionally, parity substantially modifies the risks of BMI for many outcomes; for example, a woman with parity 1 and a BMI of 30 has about the same risk of pregnancy induced hypertension, and a lower risk of pre‐eclampsia, compared to a nulliparous woman of BMI 20. A more nuanced understanding of the risks of BMI is needed.

## 1 Introduction

Across high income countries, almost 50% of women enter pregnancy with their body mass index (BMI) in the overweight or obese range [1, 2, 3, 4]. Gestational hypertension, pre‐eclampsia [5, 6, 7, 8, 9, 10, 11, 12, 13], gestational diabetes mellitus (GDM) [5, 6, 8, 9, 11, 13], need for induction of labour [8, 11, 12], and birth by caesarean section [5, 6, 7, 8, 9, 10, 11, 12, 13, 14] are all reported to occur more frequently in women with overweight and obesity. Additionally, infants born to women with overweight or obesity are more likely to be of higher birthweight when compared with women whose BMI is within the healthy range [5, 7, 15].

However, many adverse outcomes for which maternal BMI is reported to be a risk factor are causally interrelated, with the increased risk of some outcomes likely due in part or whole to the increased risk of others. For example, the observed increased risk of induction of labour partly reflects a woman's increased risk of pregnancy‐induced hypertension, pre‐eclampsia or GDM. Importantly, the occurrence of these identified pregnancy outcomes is impacted by maternal parity, a factor rarely presented separately in the literature.

Furthermore, the majority of published studies imply a degree of inevitability of adverse pregnancy outcomes in women with overweight or obesity. However, these reports have limitations. Firstly, outcome estimates tend to be presented as relative risks (RR) or odds ratios (OR), with women of “normal” BMI forming the comparator, rather than presenting the absolute risk (AR) or risk difference (RD). Secondly, quantifying risk using stepped BMI categories, rather than BMI as a continuous variable, can be misleading for individual women, particularly women whose BMI lies at either end of a BMI category, where the actual risk of an outcome may be over‐estimated at the lower and under‐estimated at the upper boundary [16].

Utilising a population dataset, the study aims were to:

1. accurately investigate the relationship between maternal BMI, parity, and adverse pregnancy outcomes;
2. provide an estimate of both relative and absolute risks of maternal BMI and adverse pregnancy outcomes; and
3. consider the potential contribution of unmeasured confounding to observed associations.

## 2 Materials and Methods

Under South Australian legislation, every birth in the state is notified to the Pregnancy Outcome Statistics Unit, Department of Health and Wellbeing, on the Supplementary Birth Record, which is completed by the midwife attending the birth. We accessed data for births occurring between January 1st, 2013, and December 31st, 2017, where maternal BMI was available. The reliability of the data collection process has been validated previously [17]. Ethical approval was obtained from the South Australian Department for Health and Wellbeing Human Research Ethics Committee (HREC/20/SAH/80).

Demographic variables included maternal age, parity, ethnicity, smoking status (defined as any smoking during pregnancy), and birthing hospital type (metropolitan public, metropolitan private, or country/rural hospital). Maternal BMI was calculated from the woman's height and weight measured at the first antenatal visit prior to 20 weeks' gestation by clinical staff.

Maternal outcomes were based on the state‐wide criteria outlined in the Perinatal Practice Guidelines current at the time [18], and included GDM; pregnancy induced hypertension (PIH; defined as clinically documented hypertension in pregnancy), pre‐eclampsia or eclampsia; induction of labour; caesarean birth; infant birthweight and birthweight *z*score (using INTERGROWTH‐21 standards; as infant sex was not available, the *z*score was assigned as the average of female and male *z*scores).

Data were deidentified, and as subsequent births to the same woman across the time‐period were not linked, two separate datasets were created consisting of women with parity 0 and parity 1 to ensure independent observations were not influenced by clustering. For analyses investigating potential differences in effect due to maternal parity, a year‐wise dataset (2015) was created within one calendar year, as it was considered extremely unlikely that a woman would have more than one birth in a 12‐month period.

### 2.1 Statistical Analysis

All statistical analyses were performed using Stata V17 (StataCorp, College Station, TX).

### 2.2 Relationship Between Maternal BMI and Pregnancy Outcomes

We undertook fractional polynomial modelling to test for nonlinear associations between maternal BMI and pregnancy outcomes, adjusted for maternal age and smoking status. We refitted the models with 1000 bootstrap samples of 1000 observations (or 5000 observations for uncommon outcomes) to assess polynomial stability. Based on these models, linear associations were determined to be reasonable for all outcomes. For some outcomes, there was a suggestion that women with very low BMI may have increased risks relative to women of normal BMI; however, the number of women with underweight BMI was very small so the presence and type of nonlinearity could not be confidently determined.

Logistic regression models were used for dichotomous outcomes, and linear regression for continuous outcomes, to estimate the change in log‐odds or means, respectively, corresponding to an increase in (continuous) BMI. For binary outcomes, results are reported as adjusted (marginal) relative risk (aRR) and adjusted risk difference (aRD) for a 5 kg/m^2^ increase in maternal BMI; we also obtained the estimated probability of each outcome at a range of BMI values (in 2‐unit increments from 18 to 44 kg/m^2^). For continuous outcomes, the mean difference corresponding to a 5 kg/m^2^ increase in BMI is reported, and the mean at the same range of BMI values was obtained. Separate models were fitted for the parity 0 and parity 1 cohorts.

For comparison, models were fitted in which BMI was collapsed into categories specified by the World Health Organisation (underweight < 18.5 kg/m^2^; normal weight 18.5–24.9 kg/m^2^; overweight 25.0–29.9 kg/m^2^; obese class I 30.0–34.9 kg/m^2^; obese class II 35.0–39.9 kg/m^2^; and obese class III > 40.0 kg/m^2^). The risk of the outcome for each BMI category was then estimated.

As many clinical outcomes are known to vary with maternal parity, we utilised data from a single calendar year only (2015) to investigate effect modification. BMI, parity, and their interaction were included in the model, and results are reported as differences in risks or differences in means, corresponding to a 5‐unit increase in maternal BMI in each parity category.

Additional post hoc analyses were carried out to investigate whether a change in diagnostic criteria for GDM during the 2013–2017 period affected the results of the primary analyses for GDM, and whether differences in management practices between metropolitan public, country/rural, and metropolitan private hospitals affected the results of the primary analyses for induction of labour and caesarean section delivery.

### 2.3 Impact of Unmeasured Confounding

Other than maternal age and smoking, there was limited scope for adjustment for additional potential confounders. To evaluate the likelihood of any unmeasured confounding and account for possible contributions from multiple causal pathways, we utilised the ‘e‐value’ method to estimate the magnitude of association (on the relative risk scale) required to explain at least 50% of the observed effect [19].

## 3 Results

Overall, data were available for 65 285 women, with 54% in their first ongoing pregnancy, and approximately 60% birthing in metropolitan maternity units (Table 1). Approximately 46% of nulliparous women entered pregnancy with BMI above 25.0 kg/m^2^, compared with 52.2% of parous women. The mean maternal age at birth was 28.43 (Standard Deviation (SD) 5.33) years for nulliparous women, compared with 30.60 (5.02) years for parous women. Demographic characteristics for women birthing during 2015 and other single‐year datasets were similar to the overall cohort (Table S1).

**TABLE 1:** Demographic characteristics for the included 65 285 births between 2013 and 2017 for parity 0 and parity 1+ cohorts (combined BMI).

	Parity 0 (*n* = 35 323)	Parity 1 (*n* = 29 962)
Age, years^a^	28.43 (5.33)	30.60 (5.02)
Location of Birth^b^		
Metropolitan teaching hospital	21 874 (61.93)	17870 (59.64)
Metropolitan private hospital	7291 (20.64)	6320 (21.09)
Country hospital	6091 (17.24)	5625 (18.77)
Other	67 (0.19)	147 (0.49)
Early pregnancy BMI (kg/m^2^)^a^	25.93 (5.93)	26.68 (6.11)
BMI Category (kg/m^2^)^b^		
< 18.5 kg/m^2^	1169 (3.31)	710 (2.37)
18.5–24.9 kg/m^2^	17 878 (50.61)	13613 (45.43)
25.0–29.9 kg/m^2^	9155 (25.92)	8412 (28.08)
30.0–34.9 kg/m^2^	4137 (11.71)	4202 (14.02)
35.0–39.9 kg/m^2^	1836 (5.20)	1900 (6, 34)
> = 40.0 kg/m^2^	1148 (5.33)	1125 (5.02)
Any smoking in pregnancy^b^	3813 (10.79)	2865 (9.56)

^a^ Mean and (Standard Deviation).

^b^ Number and percentage.

### 3.1 Relationships Between Maternal BMI and Pregnancy Outcomes

**FIGURE 1:**
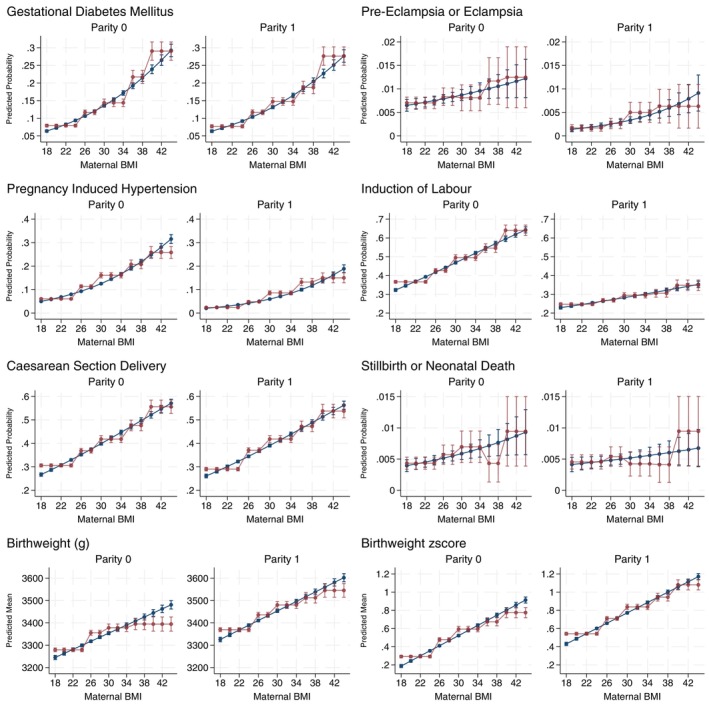
Linear representation of estimated risk of pregnancy outcome at each BMI value (continuous; blue) versus BMI categories (red) for nulliparous pregnancies compared to multiparous pregnancies.

The use of BMI categories rather than as a continuous measure resulted in overestimation of absolute risk at the lower end, and underestimation at the higher end, of each maternal BMI category (Table 2; Figure 1).

**TABLE 2:** Estimated absolute risk (AR) of pregnancy outcome at each BMI value (continuous) versus BMI categories (normal, overweight, obese) for nulliparous pregnancies compared to multiparous pregnancies.

Outcome	BMI kg/m^2^	Estimated absolute risk (AR) (95% CI) for BMI continuous	BMI category	Estimated absolute risk (AR) (95% CI) for BMI category
Nulliparous (parity 0)				
Gestational Diabetes Mellitus	18	0.064 (0.060, 0.067)	18.5–24.9	0.079 (0.075, 0.083)
	20	0.073 (0.069, 0.076)		
	22	0.083 (0.079, 0.086)		
	24	0.094 (0.091, 0.097)		
	26	0.106 (0.103, 0.110)	25.0–29.9	0.117 (0.110, 0.123)
	28	0.120 (0.117, 0.124)		
	30	0.136 (0.132, 0.140)	30.0–34.9	0.144 (0.133, 0.155)
	32	0.153 (0.148, 0.158)		
	34	0.172 (0.165, 0.178)		
	36	0.192 (0.184, 0.201)	35.0–39.9	0.217 (0.199, 0.236)
	38	0.215 (0.205, 0.225)		
	40	0.239 (0.227, 0.251)	≥ 40.0	0.291 (0.265, 0.317)
	42	0.265 (0.250, 0.280)		
	44	0.293 (0.275, 0.310)		
Pre‐Eclampsia or Eclampsia	18	0.006 (0.005, 0.008)	18.5–24.9	0.007 (0.006, 0.008)
	20	0.007 (0.006, 0.008)		
	22	0.007 (0.006, 0.008)		
	24	0.008 (0.007, 0.008)		
	26	0.008 (0.007, 0.009)	25.0–29.9	0.008 (0.006, 0.010)
	28	0.008 (0.007, 0.009)		
	30	0.009 (0.008, 0.010)	30.0–34.9	0.008 (0.005, 0.011)
	32	0.009 (0.008, 0.011)		
	34	0.010 (0.008, 0.011)		
	36	0.010 (0.008, 0.012)	35.0–39.9	0.012 (0.007, 0.017)
	38	0.011 (0.008, 0.013)		
	40	0.011 (0.008, 0.014)	≥ 40.0	0.012 (0.006, 0.019)
	42	0.012 (0.008, 0.015)		
	44	0.012 (0.008, 0.016)		
Pregnancy Induced Hypertension	18	0.050 (0.047, 0.053)	18.5–24.9	0.061 (0.057, 0.064)
	20	0.059 (0.056, 0.062)		
	22	0.068 (0.065, 0.071)		
	24	0.080 (0.077, 0.083)		
	26	0.093 (0.090, 0.096)	25.0–29.9	0.114 (0.107, 0.120)
	28	0.108 (0.105, 0.112)		
	30	0.125 (0.121, 0.129)	30.0–34.9	0.161 (0.150, 0.172)
	32	0.145 (0.140, 0.150)		
	34	0.167 (0.160, 0.173)		
	36	0.191 (0.183, 0.199)	35.0–39.9	0.207 (0.189, 0.226)
	38	0.218 (0.208, 0.229)		
	40	0.248 (0.235, 0.261)	≥ 40.0	0.258 (0.233, 0.284)
	42	0.280 (0.265, 0.296)		
	44	0.315 (0.297, 0.334)		
Induction of Labour	18	0.323 (0.315, 0.331)	18.5–24.9	0.366 (0.359, 0.374)
	20	0.346 (0.339, 0.352)		
	22	0.369 (0.363, 0.375)		
	24	0.393 (0.388, 0.398)		
	26	0.418 (0.412, 0.423)	25.0–29.9	0.427 (0.416, 0.437)
	28	0.443 (0.437, 0.448)		
	30	0.468 (0.461, 0.475)	30.0–34.9	0.495 (0.480, 0.510)
	32	0.494 (0.486, 0.501)		
	34	0.519 (0.510, 0.528)		
	36	0.545 (0.534, 0.555)	35.0–39.9	0.546 (0.523, 0.569)
	38	0.570 (0.558, 0.582)		
	40	0.595 (0.581, 0.608)	≥ 40.0	0.641 (0.613, 0.669)
	42	0.619 (0.604, 0.634)		
	44	0.643 (0.627, 0.659)		
Caesarean Section	18	0.267 (0.260, 0.274)	18.5–24.9	0.306 (0.299, 0.313)
	20	0.287 (0.281, 0.293)		
	22	0.308 (0.302, 0.313)		
	24	0.330 (0.324, 0.335)		
	26	0.352 (0.347, 0.357)	25.0–29.9	0.369 (0.359, 0.378)
	28	0.375 (0.370, 0.380)		
	30	0.399 (0.392, 0.405)	30.0–34.9	0.418 (0.403, 0.433)
	32	0.423 (0.415, 0.430)		
	34	0.447 (0.438, 0.456)		
	36	0.472 (0.461, 0.482)	35.0–39.9	0.476 (0.454, 0.499)
	38	0.497 (0.484, 0.509)		
	40	0.521 (0.508, 0.535)	≥ 40.0	0.556 (0.527, 0.584)
	42	0.546 (0.531, 0.562)		
	44	0.571 (0.554, 0.588)		
Stillbirth or Neonatal Death	18	0.004 (0.003, 0.005)	18.5–24.9	0.004 (0.003, 0.005)
	20	0.004 (0.003, 0.005)		
	22	0.005 (0.004, 0.005)		
	24	0.005 (0.004, 0.006)		
	26	0.005 (0.004, 0.006)	25.0–29.9	0.006 (0.004, 0.007)
	28	0.005 (0.005, 0.006)		
	30	0.006 (0.005, 0.007)	30.0–34.9	0.007 (0.004, 0.009)
	32	0.006 (0.005, 0.007)		
	34	0.007 (0.005, 0.008)		
	36	0.007 (0.005, 0.009)	35.0–39.9	0.004 (0.001, 0.007)
	38	0.008 (0.006, 0.010)		
	40	0.008 (0.006, 0.011)	≥ 40.0	0.009 (0.004, 0.015)
	42	0.009 (0.006, 0.012)		
	44	0.009 (0.006, 0.013)		
Birthweight (g)	18	3245.137 (3235.775, 3254.499)	18.5–24.9	3279.240 (3271.182, 3287.298)
	20	3263.264 (3255.348, 3271.181)		
	22	3281.391 (3274.665, 3288.118)		
	24	3299.519 (3293.571, 3305.466)		
	26	3317.646 (3311.896, 3323.396)	25.0–29.9	3355.437 (3344.179, 3366.695)
	28	3335.773 (3329.585, 3341.961)		
	30	3353.900 (3346.753, 3361.048)	30.0–34.9	3378.049 (3361.286, 3394.812)
	32	3372.028 (3363.576, 3380.479)		
	34	3390.155 (3380.189, 3400.121)		
	36	3408.282 (3396.673, 3419.892)	35.0–39.9	3395.021 (3369.841, 3420.202)
	38	3426.409 (3413.076, 3439.743)		
	40	3444.537 (3429.426, 3459.648)	≥ 40.0	3394.651 (3362.863, 3426.439)
	42	3462.664 (3445.739, 3479.589)		
	44	3480.791 (3462.026, 3499.556)		
Birthweight zscore	18	0.188 (0.171, 0.204)	18.5–24.9	0.292 (0.278, 0.306)
	20	0.243 (0.229, 0.257)		
	22	0.299 (0.287, 0.311)		
	24	0.355 (0.345, 0.366)		
	26	0.411 (0.401, 0.421)	25.0–29.9	0.478 (0.459, 0.498)
	28	0.467 (0.456, 0.478)		
	30	0.523 (0.510, 0.535)	30.0–34.9	0.594 (0.564, 0.623)
	32	0.579 (0.564, 0.593)		
	34	0.634 (0.617, 0.652)		
	36	0.690 (0.670, 0.711)	35.0–39.9	0.674 (0.630, 0.719)
	38	0.746 (0.723, 0.770)		
	40	0.802 (0.775, 0.829)	≥ 40.0	0.775 (0.719, 0.831)
	42	0.858 (0.828, 0.888)		
	44	0.914 (0.881, 0.947)		
Multiparous (parity 1)				
Gestational Diabetes Mellitus	18	0.063 (0.060, 0.067)	18.5–24.9	0.077 (0.073, 0.081)
	20	0.072 (0.068, 0.076)		
	22	0.081 (0.078, 0.085)		
	24	0.092 (0.088, 0.095)		
	26	0.104 (0.100, 0.107)	25.0–29.9	0.117 (0.110, 0.124)
	28	0.117 (0.113, 0.120)		
	30	0.131 (0.127, 0.135)	30.0–34.9	0.147 (0.137, 0.158)
	32	0.147 (0.142, 0.152)		
	34	0.165 (0.158, 0.171)		
	36	0.184 (0.176, 0.192)	35.0–39.9	0.187 (0.170, 0.205)
	38	0.205 (0.195, 0.215)		
	40	0.227 (0.215, 0.239)	≥ 40.0	0.277 (0.250, 0.303)
	42	0.251 (0.236, 0.266)		
	44	0.277 (0.259, 0.294)		
Pre‐Eclampsia or Eclampsia	18	0.001 (0.001, 0.002)	18.5–24.9	0.002 (0.001, 0.002)
	20	0.002 (0.001, 0.002)		
	22	0.002 (0.001, 0.002)		
	24	0.002 (0.002, 0.003)		
	26	0.003 (0.002, 0.003)	25.0–29.9	0.003 (0.002, 0.004)
	28	0.003 (0.002, 0.004)		
	30	0.003 (0.003, 0.004)	30.0–34.9	0.005 (0.003, 0.007)
	32	0.004 (0.003, 0.005)		
	34	0.004 (0.003, 0.006)		
	36	0.005 (0.004, 0.006)	35.0–39.9	0.006 (0.003, 0.010)
	38	0.006 (0.004, 0.008)		
	40	0.007 (0.005, 0.009)	≥ 40.0	0.006 (0.002, 0.011)
	42	0.008 (0.005, 0.011)		
	44	0.009 (0.005, 0.013)		
Pregnancy Induced Hypertension	18	0.020 (0.019, 0.022)	18.5–24.9	0.024 (0.021, 0.026)
	20	0.025 (0.022, 0.027)		
	22	0.029 (0.027, 0.032)		
	24	0.035 (0.033, 0.037)		
	26	0.042 (0.040, 0.044)	25.0–29.9	0.048 (0.044, 0.053)
	28	0.050 (0.048, 0.053)		
	30	0.060 (0.057, 0.063)	30.0–34.9	0.086 (0.078, 0.095)
	32	0.071 (0.068, 0.075)		
	34	0.084 (0.080, 0.089)		
	36	0.100 (0.094, 0.106)	35.0–39.9	0.132 (0.117, 0.148)
	38	0.118 (0.110, 0.126)		
	40	0.138 (0.128, 0.149)	≥ 40.0	0.150 (0.129, 0.171)
	42	0.162 (0.149, 0.175)		
	44	0.189 (0.172, 0.205)		
Induction of Labour	18	0.229 (0.221, 0.237)	18.5–24.9	0.247 (0.240, 0.255)
	20	0.237 (0.231, 0.244)		
	22	0.246 (0.240, 0.252)		
	24	0.255 (0.249, 0.260)		
	26	0.264 (0.259, 0.269)	25.0–29.9	0.268 (0.259, 0.277)
	28	0.273 (0.268, 0.278)		
	30	0.282 (0.276, 0.288)	30.0–34.9	0.295 (0.281, 0.308)
	32	0.292 (0.285, 0.299)		
	34	0.302 (0.293, 0.310)		
	36	0.311 (0.302, 0.321)	35.0–39.9	0.305 (0.285, 0.326)
	38	0.322 (0.310, 0.333)		
	40	0.332 (0.319, 0.345)	≥ 40.0	0.348 (0.320, 0.376)
	42	0.342 (0.327, 0.357)		
	44	0.353 (0.336, 0.370)		
Caesarean Section	18	0.261 (0.253, 0.269)	18.5–24.9	0.290 (0.283, 0.298)
	20	0.281 (0.274, 0.288)		
	22	0.301 (0.295, 0.308)		
	24	0.323 (0.317, 0.328)		
	26	0.345 (0.339, 0.350)	25.0–29.9	0.371 (0.360, 0.381)
	28	0.367 (0.362, 0.373)		
	30	0.391 (0.384, 0.397)	30.0–34.9	0.418 (0.403, 0.433)
	32	0.415 (0.407, 0.422)		
	34	0.439 (0.430, 0.448)		
	36	0.464 (0.453, 0.474)	35.0–39.9	0.472 (0.450, 0.494)
	38	0.488 (0.476, 0.501)		
	40	0.513 (0.499, 0.527)	≥ 40.0	0.538 (0.509, 0.566)
	42	0.538 (0.522, 0.554)		
	44	0.562 (0.545, 0.580)		
Stillbirth or Neonatal Death	18	0.004 (0.003, 0.005)	18.5–24.9	0.005 (0.003, 0.006)
	20	0.004 (0.003, 0.005)		
	22	0.004 (0.004, 0.005)		
	24	0.005 (0.004, 0.005)		
	26	0.005 (0.004, 0.006)	25.0–29.9	0.005 (0.004, 0.007)
	28	0.005 (0.004, 0.006)		
	30	0.005 (0.004, 0.006)	30.0–34.9	0.004 (0.002, 0.006)
	32	0.005 (0.004, 0.006)		
	34	0.006 (0.004, 0.007)		
	36	0.006 (0.004, 0.007)	35.0–39.9	0.004 (0.001, 0.007)
	38	0.006 (0.004, 0.008)		
	40	0.006 (0.004, 0.008)	≥ 40.0	0.009 (0.004, 0.015)
	42	0.007 (0.004, 0.009)		
	44	0.007 (0.004, 0.010)		
Birthweight (g)	18	3325.581 (3315.539, 3335.622)	18.5–24.9	3369.562 (3360.773, 3378.352)
	20	3346.877 (3338.328, 3355.426)		
	22	3368.174 (3360.901, 3375.447)		
	24	3389.470 (3383.125, 3395.815)		
	26	3410.767 (3404.836, 3416.698)	25.0–29.9	3435.875 (3424.701, 3447.050)
	28	3432.064 (3425.928, 3438.199)		
	30	3453.360 (3446.457, 3460.264)	30.0–34.9	3480.068 (3464.240, 3495.896)
	32	3474.657 (3466.580, 3482.734)		
	34	3495.954 (3486.448, 3505.459)		
	36	3517.250 (3506.158, 3528.343)	35.0–39.9	3512.484 (3488.949, 3536.018)
	38	3538.547 (3525.769, 3551.325)		
	40	3559.844 (3545.315, 3574.371)	≥ 40.0	3545.430 (3514.857, 3576.003)
	42	3581.140 (3564.818, 3597.462)		
	44	3602.437 (3584.290, 3620.583)		
Birthweight zscore	18	0.431 (0.413, 0.449)	18.5–24.9	0.543 (0.527, 0.559)
	20	0.488 (0.472, 0.503)		
	22	0.545 (0.531, 0.558)		
	24	0.602 (0.590, 0.613)		
	26	0.659 (0.648, 0.669)	25.0–29.9	0.710 (0.690, 0.731)
	28	0.715 (0.704, 0.727)		
	30	0.772 (0.760, 0.785)	30.0–34.9	0.838 (0.809, 0.866)
	32	0.829 (0.815, 0.844)		
	34	0.886 (0.869, 0.904)		
	36	0.943 (0.923, 0.963)	35.0–39.9	0.945 (0.902, 0.988)
	38	1.000 (0.977, 1.023)		
	40	1.057 (1.031, 1.083)	≥ 40.0	1.080 (1.025, 1.136)
	42	1.114 (1.084, 1.144)		
	44	1.171 (1.138, 1.204)		

### 3.2 Relationship Between Maternal BMI and GDM

A 5 kg/m^2^ increase in maternal BMI was associated with an adjusted relative risk (aRR) of GDM between 1.3 and 1.4 times (Parity 0:aRR 1.37, 95% CI 1.34–1.40; Parity 1:aRR 1.35, 95% CI 1.321.39), with an adjusted risk difference (aRD) of approximately 3% (Parity 0:aRD 3.05%, 95% CI: 2.86–3.25; Parity 1:aRD 2.87%, 95% CI 2.68–3.07; Table 3). There was no evidence of effect modification by parity. The absolute risk of GDM ranged from approximately 7%–15% at BMI 20–32 kg/m^2^, respectively (Table 2).

**TABLE 3:** Adjusted estimates for effect of BMI on pregnancy outcomes, for nulliparous pregnancies compared to multiparous pregnancies.

Outcome	BMI			Contrast	Adjusted Risk Difference	Adjusted Relative Risk	E Value
	22 kg/m^*2*^	27 kg/m^2^	32 kg/m^2^		aRD (95% CI)	aRR (95% CI)	
Nulliparous (parity 0)							
Gestational Diabetes Mellitus	7.58	11.34	15.56	27v22	3.054 (2.858, 3.249)	1.370 (1.339, 1.401)	1.58
				32v27	3.982 (3.661, 4.302)	1.352 (1.324, 1.380)	1.56
Pre‐Eclampsia or Eclampsia	0.62	0.94	1.26	27v22	0.093 (0.026, 0.159)	1.129 (1.032, 1.236)	1.31
				32v27	0.104 (0.021, 0.188)	1.129 (1.032, 1.235)	1.31
Pregnancy Induced Hypertension	6.81	12.34	17.05	27v22	3.190 (3.017, 3.363)	1.466 (1.432, 1.501)	1.66
				32v27	4.443 (4.129, 4.758)	1.443 (1.412, 1.474)	1.64
Induction of Labour	36.98	43.73	47.48	27v22	6.121 (5.686, 6.556)	1.166 (1.153, 1.179)	1.36
				32v27	6.342 (5.880, 6.804)	1.147 (1.137, 1.158)	1.34
Caesarean Birth	30.88	39.27	42.56	27v22	5.550 (5.150, 5.949)	1.180 (1.166, 1.195)	1.38
				32v27	5.928 (5.476, 6.380)	1.163 (1.151, 1.175)	1.36
Birthweight (g)	3304.99	3348.62	3373.57	27v22	45.318 (40.460, 50.177)	—	—
				32v27	45.318 (40.460, 50.177)	—	—
Birthweight z‐score	0.34	0.45	0.61	27v22	0.140 (0.131, 0.148)	—	—
				32v27	0.140 (0.131, 0.148)	—	—
Multiparous (parity 1+)							
Gestational Diabetes Mellitus	7.59	11.92	16.70	27v22	2.874 (2.677, 3.072)	1.354 (1.321, 1.387)	1.57
				32v27	3.716 (3.393, 4.038)	1.338 (1.308, 1.368)	1.55
Pre‐Eclampsia or Eclampsia	0.19	0.12	0.57	27v22	0.081 (0.056, 0.106)	1.434 (1.258, 1.634)	1.64
				32v27	0.116 (0.068, 0.165)	1.433 (1.258, 1.633)	1.64
Pregnancy Induced Hypertension	2.17	4.89	8.64	27v22	1.654 (1.550, 1.757)	1.563 (1.512, 1.616)	1.74
				32v27	2.517 (2.304, 2.729)	1.548 (1.499, 1.598)	1.73
Induction of Labour	24.79	28.44	29.55	27v22	2.230 (1.850, 2.611)	1.091 (1.074, 1.108)	1.26
				32v27	2.354 (1.930, 2.778)	1.088 (1.072, 1.104)	1.25
Caesarean Section	29.28	37.59	44.20	27v22	5.476 (5.064, 5.888)	1.182 (1.166, 1.198)	1.38
				32v27	5.870 (5.400, 6.341)	1.165 (1.152, 1.178)	1.36
Birthweight (g)	3382.82	3430.84	3451.62	27v22	53.242 (48.383, 58.100)	—	—
				32v27	53.242 (48.383, 58.100)	—	—
Birthweight z‐score	0.58	0.70	0.79	27v22	0.142 (0.134, 0.151)	—	—
				32v27	0.142 (0.134, 0.151)	—	—

Year of birth (as a proxy for the diagnostic criteria used for GDM) was not a confounder for the effect of BMI on risk of GDM, although rates of GDM diagnosis increased after the change of criteria. However, some evidence of effect modification was observed: the additional risk associated with increased BMI was greater after the adoption of the new diagnostic criteria. (data not presented).

### 3.3 Relationship Between Maternal BMI and Pre‐Eclampsia or Eclampsia

A 5 kg/m^2^ increase in maternal BMI was associated with an aRR of pre‐eclampsia or eclampsia in nulliparous women of 1.13 times (aRR 1.13, 95% CI: 1.03 to 1.24 times) increasing to 1.43 times (aRR 1.43, 95% CI: 1.26, 1.63 times) in parous women. However, the aRD was less than 0.1% in both cohorts (parity 0: 0.09%, 95% CI: 0.03 to 0.16; parity 1: 0.08%, 95% CI: 0.06 to 0.11; Table 3). The absolute risk of pre‐eclampsia or eclampsia in nulliparous women ranged from 0.7% (95% CI: 0.6 to 0.8) at BMI 20 kg/m^2^, to 0.9% (95% CI: 0.8 to 1.1) at BMI 32 kg/m^2^, while in parous women, the absolute risk ranged from 0.2% (95% CI: 0.1 to 0.2) to 0.4% (95% CI: 0.3 to 0.5%; Table 2). There was no evidence of effect modification by parity, but the risk of pre‐eclampsia or eclampsia was lower at parity 1 across all maternal BMI values.

### 3.4 Relationship Between Maternal BMI and Pregnancy Induced Hypertension

A 5 kg/m^2^ increase in maternal BMI was associated with an approximately 1.5 to 1.6 times aRR of pregnancy related hypertension. However, the aRD in parous women (aRD 1.65; 95% CI 1.55 to 1.76%) was substantially lower than that in nulliparous women (aRD 3.19; 95% CI 3.02 to 3.36%) (Table 3), such that the absolute risk in parous women with BMI 30 kg/m^2^ (AR 0.6%; 95% CI: 0.57 to 0.63) was similar to that in nulliparous women with BMI 20 kg/m^2^ (AR 0.6%; 95% CI: 0.56 to 0.62; Table 2).

### 3.5 Relationship Between Maternal BMI and Induction of Labour

The relationship between maternal BMI and induction of labour varied with parity, such that the association was stronger in nulliparous women particularly at higher BMI values, and lower in parous women. For nulliparous women, the aRR for induction of labour was 1.17 (95% CI 1.15 to 1.18), while the aRD for a 5 kg/m^2^ increase in BMI was 6.12% (95% CI 5.69 to 6.56; Table 3). For nulliparous women, the absolute risk of induction of labour increased from 35% at a BMI of 20 kg/m^2^ to 49% at a BMI of 32 kg/m^2^, compared with 24% at a BMI of 20 kg/m^2^ and 29% at a BMI of 32 kg/m^2^ in parous women (Table 2).

Post hoc analyses investigated the relationship between hospital type (metropolitan public, metropolitan private, and country hospital), BMI and induction of labour. Hospital type was not a confounder of the relationships between BMI and caesarean birth, although there was some evidence of effect modification (data not presented).

### 3.6 Relationship Between Maternal BMI and Caesarean Birth

A 5 kg/m^2^ increase in maternal BMI was associated with an aRR of caesarean birth of approximately 1.18 times (Parity 0 and Parity 1: aRR 1.18, 95% CI 1.17 to 1.20), with aRD similar among multiparous and nulliparous women (approximately 5.48% and 5.55% respectively; Table 3). The absolute risk of caesarean birth was similar for parous compared to nulliparous women, ranging from 28%–42% at BMI 20–32 kg/m^2^ respectively (Table 2).

As with induction of labour, post hoc analyses showed some evidence of effect modification by hospital type (data not presented).

### 3.7 Relationship Between Maternal BMI and Birthweight

A 5 kg/m^2^ increase in BMI was associated with an average increase of approximately 50 g in birthweight. Regardless of maternal BMI, infant birthweight for parous women was approximately 80 g higher than for nulliparous women (Table 3).

### 3.8 Relationship Between Maternal BMI and Birthweight *z*Score

A 5 kg/m^2^ increase in maternal BMI was associated with an increase of approximately 0.14 standard deviations in birthweight *z*score and was similar across parity cohorts. However, the estimated effect of parity was greater than that of increasing maternal BMI, increasing birthweight *z*score by approximately 0.25 SD for parous women across all BMI values (Table 3).

### 3.9 Relationship Between Maternal BMI and Perinatal Mortality

The relationship between maternal BMI and perinatal mortality was unstable across parity groups, reflecting its infrequent occurrence, even in this large dataset (Table 2).

### 3.10 Effect of Unmeasured Confounding

For antenatal outcomes, unmeasured confounding on a relative risk scale of approximately 1.6 times (i.e., the confounder confers a 1.6 times higher risk of increased BMI, and a 1.6 times higher risk of the outcome) would be sufficient to explain 50% of the observed association between BMI and outcomes (Table 3). For infant outcomes, weaker levels of confounding would be sufficient to explain 50% of the observed association. Due to the large sample size, the e‐value for the confidence bound closest to the null was generally similar to the e‐value for the point estimate (Table 3).

## 4 Discussion

Our findings highlight that the use of maternal BMI categories to determine the risk of pregnancy outcomes can be misleading. Specifically, risk is over‐estimated at the lower end and under‐estimated at the upper end of each BMI category. This generates estimates which do not reveal the true risk for many women, reflecting loss of statistical power and considerable variability in risk across each category descriptor.

Secondly, our findings demonstrate the value of using absolute risk for adverse pregnancy outcomes, and the need to consider the impact of both maternal BMI and parity. For example, there is an increasing risk for GDM of approximately 37% with increasing maternal BMI. However, the absolute risk is more modest, increasing from approximately 7% for women with BMI 20 kg/m^2^ to 15% for women with BMI 32 kg/m^2^, with little evidence of effect modification by parity. In contrast, the absolute risk of pre‐eclampsia is largely related to maternal parity, with the risk for a parous woman with BMI 32 kg/m^2^ similar to the risk for a nulliparous woman with BMI 20 kg/m^2^. The risk of pregnancy induced hypertension, similarly, is virtually the same for a parous women with BMI 30 kg/m^2^ and a nulliparous woman with BMI 20 kg/m^2^. This suggests that counselling regarding the risks of increased BMI should be quite different for women planning subsequent pregnancies.

Finally, we note that the estimated effect of BMI on outcomes is the total effect, i.e., via all causal pathways. Alongside assumed biological mechanisms, there may be other causal mechanisms involved, notably including clinician decisions based on the perception of risk associated with higher BMI. Given the significant weight bias encountered by women within higher BMI categories, it is pertinent to consider these other factors in addition to the woman's BMI, both when conveying risk and considering recommendations for care.

Our findings of an increased relative risk of adverse pregnancy outcomes with increasing maternal BMI are consistent with the extensive literature as previously outlined [5, 6, 7, 8, 9, 10, 11, 12, 13]. However, a significant point of difference is the presentation of absolute risks and inclusion of maternal parity in addition to BMI.

How risk is perceived by healthcare providers and women varies [20]. Perception of risk will be influenced by an individual's health literacy, numeracy and understanding of probability [21], preferences around information presented in a written, verbal or pictorial format [22], and their social, emotional, and cultural environment [21, 23]. For healthcare professionals, presentation of risk is often considered to be objective, although influenced by practitioner numeracy [23]. While healthcare providers often present information as a relative risk, use of relative risk, odds ratios and absolute risk is often interchangeable and differences in the estimates are blurred [23].

Many professional colleges recommend pre‐conception counselling for women with a higher BMI regarding the increased risks associated with obesity in pregnancy, and recommendations to “lose weight” prior to pregnancy to avert adverse outcomes [24, 25]. Blunt assessments of “risk” have resulted in policies and procedures which subsequently limit choices for a woman's pregnancy and birth care, further reinforcing weight stigma and individual responsibility for obesity [26, 27].

Keely and colleagues [28] found that women may acknowledge risks associated with obesity in pregnancy, though most “try not to think about them”, or reject connections between pregnancy complications and weight. Furthermore, Feltham and colleagues [26] identified that women expressed little understanding or perception of risk associated with obesity in pregnancy. These reports are consistent with our previous findings, highlighting that women are often unable to articulate specific risks or harms for themselves or their infant, in relation to the impact of obesity [29, 30, 31]. Women who did recognise the potential health impacts associated with obesity state this was not their “main motivator” to initiate behavioural changes [30].

The population registry utilised in this study has some limitations. These include a lack of information on potential confounders, such as ethnicity and socioeconomic status, which are known to be related both to maternal BMI and adverse pregnancy outcomes. The e‐values calculated for many outcomes are of a plausible magnitude for confounding relationships, meaning that unmeasured confounding cannot be ruled out as an explanation for the observed results. Additionally, we are unable to link the same mother across subsequent pregnancies, meaning that we could only conduct analyses either within a single parity category or within a single year, in order to ensure all observations were independent. However, the strengths of these data include the many years it has been recorded, with birth attendants well versed in completing the data collection, and its inclusivity of all births in South Australia, eliminating bias from selective data collection. While we did not identify evidence of confounding by hospital type, and relationships between BMI and both induction of labour and caesarean birth, there was evidence of effect modification. This finding requires additional evaluation and is beyond the scope of this current manuscript.

## 5 Conclusions

We would argue that the simple use of relative risk estimates within BMI categories overinflates the perceived risk of maternal BMI on pregnancy complications, and that the use of BMI categories leads to incorrect estimates even of relative risks. Collaborative care and improved pregnancy and birth outcomes for women and infants are contingent on a greater understanding of absolute risks and the impact from other confounding factors as well as effect modifiers, notably parity. Maintaining an approach where healthcare discussions and policies are based on a broad presentation of relative risk estimates will continue to limit maternity care choices for women, further reinforcing individual responsibility for obesity and experiences of weight stigma. A more nuanced and individualised discussion is required.

## Funding

National Health and Medical Research Council (1196133).

## Conflicts of Interest

The authors declare no conflicts of interest.

## Supporting information


**Table S1:** Baseline characteristics by year for all birth by year 2013–2017.

## Data Availability

Data are available from Preventative Health SA, following application of a data request via the Preventative Health SA website (https://www.preventivehealth.sa.gov.au/evidence‐data/research‐requests) and appropriate ethical approval.
