# Supplementary material for: What Is the Contribution of Maternal BMI to the Risk of Adverse Pregnancy Outcomes?
Source: Aust N Z J Obstet Gynaecol. 2026 Jul 27;66(4):e70163. doi: 10.1111/ajo.70163 (PMC13408330; doi:10.1111/ajo.70163)
Supplement: Supplementary file 1 — Table S1: Baseline characteristics by year for all birth by year 2013–2017. [file AJO-66-0-s001.docx]

# Supplementary Table 1: Baseline characteristics by year for all birth by year 2013-2017

| **Characteristic** | **2013** | **2014** | **2015** | **2016** | **2017** |
| --- | --- | --- | --- | --- | --- |
| Overall Numbers | n=15973 | n=16951 | n=16722 | n=16937 | n=16626 |
| BMI Category: N(%) |  |  |  |  |  |
| - < 18.5 kg/m^2^ | 433 (2.71) | 464 (2.74) | 471 (2.82) | 434 (2.56) | 402 (2.42) |
| - 18.5-24.9 kg/m^2^ | 7367 (46.12) | 7741 (45.67) | 7618 (45.56) | 7799 (46.05) | 7649 (46.01) |
| - 25.0-29.9 kg/m^2^ | 4375 (27.39) | 4631 (27.32) | 4642 (27.76) | 4655 (27.48) | 4520 (27.19) |
| - 30.0-34.9 kg/m^2^ | 2196 (13.75) | 2422 (14.29) | 2287 (13.68) | 2249 (13.28) | 2344 (14.10) |
| - 35.0-39.9 kg/m^2^ | 986 (6.17) | 1045 (6.16) | 1075 (6.43) | 1067 (6.30) | 1054 (6.34) |
| - ≥ 40.0 kg/m^2^ | 616 (3.86) | 648 (3.82) | 629 (3.76) | 733 (4.33) | 657 (3.95) |
| BMI (kg/m^2^): Mean (SD) | 26.57 (6.14) | 26.63 (6.16) | 26.60 (6.13) | 26.65 (6.26) | 26.67 (6.18) |
| Smoked in Pregnancy: N(%) |  |  |  |  |  |
| - No | 13724 (85.92) | 14799 (87.30) | 14675 (87.76) | 14977 (88.43) | 14838 (89.25) |
| - Yes | 2211 (13.84) | 2108 (12.44) | 1986 (11.88) | 1925 (11.37) | 1734 (10.43) |
| - Missing | 38 (0.24) | 44 (0.26) | 61 (0.36) | 35 (0.21) | 54 (0.32) |
| Age (years): Mean (SD) | 29.63 (5.46) | 29.86 (5.42) | 29.98 (5.33) | 30.18 (5.27) | 30.31 (5.20) |
| Parity Category: N(%) |  |  |  |  |  |
| - 0 | 7032 (44.02) | 7211 (42.54) | 7009 (41.91) | 7029 (41.50) | 7042 (42.36) |
| - 1 | 5526 (34.60) | 6045 (35.66) | 6117 (36.58) | 6266 (37.00) | 6008 (36.14) |
| - 2+ | 3415 (21.38) | 3695 (21.80) | 3596 (21.50) | 3642 (21.50) | 3576 (21.51) |
| Hospital Type: N(%) |  |  |  |  |  |
| - Metro Public | 9748 (61.03) | 10301 (60.77) | 10293 (61.55) | 10417 (61.50) | 10337 (62.17) |
| - Country | 3082 (19.30) | 3294 (19.43) | 3185 (19.05) | 3271 (19.31) | 3179 (19.12) |
| - Metro Private | 3066 (19.19) | 3282 (19.36) | 3164 (18.92) | 3176 (18.75) | 3043 (18.30) |
| - Home | 77 (0.48) | 74 (0.44) | 80 (0.48) | 73 (0.43) | 67 (0.40) |

| Stillbirth or Neonatal Death | 18 | 0.004 (0.003, 0.005) | 18.5-24.9 | 0.004 (0.003, 0.005) |
| --- | --- | --- | --- | --- |
|  | 20 | 0.004 (0.003, 0.005) |  |  |
|  | 22 | 0.005 (0.004, 0.005) |  |  |
|  | 24 | 0.005 (0.004, 0.006) |  |  |
|  | 26 | 0.005 (0.004, 0.006) | 25.0-29.9 | 0.006 (0.004, 0.007) |
|  | 28 | 0.005 (0.005, 0.006) |  |  |
|  | 30 | 0.006 (0.005, 0.007) | 30.0-34.9 | 0.007 (0.004, 0.009) |
|  | 32 | 0.006 (0.005, 0.007) |  |  |
|  | 34 | 0.007 (0.005, 0.008) |  |  |
|  | 36 | 0.007 (0.005, 0.009) | 35.0-39.9 | 0.004 (0.001, 0.007) |
|  | 38 | 0.008 (0.006, 0.010) |  |  |
|  | 40 | 0.008 (0.006, 0.011) | ≥40.0 | 0.009 (0.004, 0.015) |
|  | 42 | 0.009 (0.006, 0.012) |  |  |
|  | 44 | 0.009 (0.006, 0.013) |  |  |
| Birthweight (g) | 18 | 3245.137 (3235.775, 3254.499) | 18.5-24.9 | 3279.240 (3271.182, 3287.298) |
|  | 20 | 3263.264 (3255.348, 3271.181) |  |  |
|  | 22 | 3281.391 (3274.665, 3288.118) |  |  |
|  | 24 | 3299.519 (3293.571, 3305.466) |  |  |
|  | 26 | 3317.646 (3311.896, 3323.396) | 25.0-29.9 | 3355.437 (3344.179, 3366.695) |
|  | 28 | 3335.773 (3329.585, 3341.961) |  |  |
|  | 30 | 3353.900 (3346.753, 3361.048) | 30.0-34.9 | 3378.049 (3361.286, 3394.812) |
|  | 32 | 3372.028 (3363.576, 3380.479) |  |  |
|  | 34 | 3390.155 (3380.189, 3400.121) |  |  |
|  | 36 | 3408.282 (3396.673, 3419.892) | 35.0-39.9 | 3395.021 (3369.841, 3420.202) |
|  | 38 | 3426.409 (3413.076, 3439.743) |  |  |
|  | 40 | 3444.537 (3429.426, 3459.648) | ≥40.0 | 3394.651 (3362.863, 3426.439) |
|  | 42 | 3462.664 (3445.739, 3479.589) |  |  |
|  | 44 | 3480.791 (3462.026, 3499.556) |  |  |
| Birthweight zscore | 18 | 0.188 (0.171, 0.204) | 18.5-24.9 | 0.292 (0.278, 0.306) |
|  | 20 | 0.243 (0.229, 0.257) |  |  |
|  | 22 | 0.299 (0.287, 0.311) |  |  |
|  | 24 | 0.355 (0.345, 0.366) |  |  |
|  | 26 | 0.411 (0.401, 0.421) | 25.0-29.9 | 0.478 (0.459, 0.498) |
|  | 28 | 0.467 (0.456, 0.478) |  |  |
|  | 30 | 0.523 (0.510, 0.535) | 30.0-34.9 | 0.594 (0.564, 0.623) |
|  | 32 | 0.579 (0.564, 0.593) |  |  |
|  | 34 | 0.634 (0.617, 0.652) |  |  |
|  | 36 | 0.690 (0.670, 0.711) | 35.0-39.9 | 0.674 (0.630, 0.719) |
|  | 38 | 0.746 (0.723, 0.770) |  |  |
|  | 40 | 0.802 (0.775, 0.829) | ≥40.0 | 0.775 (0.719, 0.831) |
|  | 42 | 0.858 (0.828, 0.888) |  |  |
|  | 44 | 0.914 (0.881, 0.947) |  |  |
